# Assessing and Optimizing Socio-Moral Reasoning Skills: Findings From the MorALERT Serious Video Game

**DOI:** 10.3389/fpsyg.2021.767596

**Published:** 2022-01-21

**Authors:** Hamza Zarglayoun, Juliette Laurendeau-Martin, Ange Tato, Evelyn Vera-Estay, Aurélie Blondin, Arnaud Lamy-Brunelle, Sameh Chaieb, Frédérick Morasse, Aude Dufresne, Roger Nkambou, Miriam H. Beauchamp

**Affiliations:** ^1^Department of Psychology, University of Montreal, Montreal, QC, Canada; ^2^Sainte-Justine Hospital Research Center, Montreal, QC, Canada; ^3^Department of Computer Science, Université du Québec à Montréal, Montreal, QC, Canada; ^4^School of Psychology, Pontificia Universidad Católica de Chile, Santiago, Chile; ^5^Department of Communication, University of Montreal, Montreal, QC, Canada

**Keywords:** moral reasoning, serious video games, adolescence, empathy, presence, neuropsychology, assessment, intervention

## Abstract

**Background:**

Social cognition and competence are a key part of daily interactions and essential for satisfying relationships and well-being. Pediatric neurological and psychological conditions can affect social cognition and require assessment and remediation of social skills. To adequately approximate the complex and dynamic nature of real-world social interactions, innovative tools are needed. The aim of this study was to document the performance of adolescents on two versions of a serious video game presenting realistic, everyday, socio-moral conflicts, and to explore whether their performance is associated with empathy or sense of presence, factors known to influence social cognition.

**Methods:**

Participants (12–17 years, *M* = 14.39; *SD* = 1.35) first completed a pre-test measure of socio-moral reasoning based on three dilemmas from a previously validated computer task. Then, they either played an evaluative version (*n* = 24) or an adaptive (*n* = 33) version of a video game presenting nine social situations in which they made socio-moral decisions and provided justifications. In the evaluative version, participants’ audio justifications were recorded verbatim and coded manually to obtain a socio-moral reasoning maturity score. In the adaptive version (AV), tailored feedback and social reinforcements were provided based on participant responses. An automatic coding algorithm developed using artificial intelligence was used to determine socio-moral maturity level in real-time and to provide a basis for the feedback and reinforcements in the game. All participants then completed a three-dilemma post-test assessment.

**Results:**

Those who played the adaptive version showed improved SMR across the pre-test, in-game and post-test moral maturity scores, *F*(1.97,63.00) = 9.81, *p_HF_* < 0.001, ϵ^2^ = 0.21, but those who played the Evaluative version did not. Socio-moral reasoning scores from both versions combined did not correlate with empathy or sense of presence during the game, though results neared significance. The study findings support preliminary validation of the game as a promising method for assessing and remediating social skills during adolescence.

## Introduction

Daily socio-emotional interactions play an important role in shaping the social brain, especially during childhood and adolescence (Blakemore, 2012; Immordino-Yang et al., 2019). In parallel, the emergence and maturation of socio-cognitive skills supports the ability to create bonds, be aware and understand social situations, and make decisions according to context and societal norms (Beauchamp and Anderson, 2010). Socio-moral reasoning (SMR) is an important socio-cognitive building block defined as the ability to analyze social situations according to moral criteria in order to distinguish right from wrong and regulate behavior in everyday life (Haidt, 2001; Gibbs, 2014). Sound SMR is associated with prosocial behavior, altruistic personality traits and overall better social competence (Eisenberg et al., 2002; Malti et al., 2009; Malti and Latzko, 2010). Conversely, SMR impairments have been linked to maladaptive behaviors, including aggression, rule-breaking and criminality (Arsenio and Lemerise, 2004; Stams et al., 2006; Van Vugt et al., 2011).

### Socio-Cognitive Development During Adolescence

Adolescence is an important period for social maturation given the increased autonomy, social network complexity, as well as environmental, biological, neural and cognitive changes that characterize this period (Blakemore, 2012). Environmentally, adolescents tend to reduce their reliance on parents, focus on peer relationships and start modeling them to fit in (Clausen, 1991; Harris, 1995). Peer opinion plays a major role in social decision-making as they become more sensitive to the approval of others and experiment new social roles (Jones et al., 2014). Biologically, pubertal hormones bring about changes that influence how adolescents interact with their peers and surroundings, as well as a strong drive for reward seeking (Sato et al., 2008; Blakemore et al., 2010). Neurally, synaptic pruning and myelination in prefrontal regions continues into late adolescence, leading to more efficient cognitive processing and behavioral changes as well as an eventual reduction in risky behavior tendencies and expanded inhibitory capacities for refraining from inappropriate social behavior (Casey et al., 2005; Blakemore, 2008; Blakemore and Mills, 2014; Qu et al., 2015). Cognitively, executive and socio-cognitive functions such as affect recognition, theory of mind, and empathy develop in parallel allowing flexible processing of complex social stimuli (Tousignant et al., 2017; Beaudoin and Beauchamp, 2020). Thus, both experience and biology underpin the socio-cognitive foundations that promote SMR maturation (Vera-Estay et al., 2015, 2016; Beaudoin and Beauchamp, 2020). Viewed from a cognitive-developmental perspective, SMR development is depicted as a progression from egocentric viewpoints to internalization of societal values throughout childhood and adolescence (Gibbs, 2014). Children and adolescents also learn to distinguish moral, social and psychological knowledge related to moral issues such as fairness, justice, welfare and rights (Turiel, 2002; Killen et al., 2011).

### Empathy and Socio-Moral Reasoning

Alongside SMR, empathy also undergoes protracted development during adolescence (Tousignant et al., 2017). Empathy consists of two primary components: an affective response to another person (sharing another’s emotional state) and a cognitive component enabling perspective taking while maintaining a self-other distinction (Jackson et al., 2005). Since SMR in part depends on emotional state (Miller et al., 1996; Zarinpoush et al., 2000), it is posited that empathic tendencies contribute to SMR and decision-making. However, studies to date report mixed findings regarding such an association. Some report a positive correlation between SMR and empathy functions (Hoffman, 2001; Dooley et al., 2010; Vera-Estay et al., 2016; Morasse et al., 2021), while others suggest that empathy can, in some cases, lead to amoral behaviors due to partiality that can cloud moral judgment (Batson et al., 1995; Decety and Cowell, 2014). For example, it may be more difficult to maintain a moral stance when a family member or someone we identify with is in a dire situation. Thus, associations between empathy and SMR need to be clarified.

### Socio-Moral Reasoning Difficulties in Adolescence

A number of risk factors impede optimal SMR maturation. Identifying and remediating putative SMR difficulties is thus essential during adolescence. Neurodevelopmental and acquired brain conditions such as Traumatic Brain Injury (Beauchamp et al., 2019), Autism Spectrum Disorders (Moran et al., 2011; Senland and Higgins-D’Alessandro, 2013) and Schizophrenia (Abdi and Sharma, 2004) have been associated with altered SMR. Environmental factors and psychological conditions can also constitute risk factors for altered social development and poor SMR, such as socioeconomic status (Bradley and Corwyn, 2002) or behavioral problems (Nelson et al., 1990; Stams et al., 2006) and may require remediation or rehabilitation. Conventional methods of intervention often take the form of cognitive development programs (e.g., moral dilemma discussion sessions) for high-risk adolescents with behavior disorders (Arbuthnot and Gordon, 1986) and moral reasoning promotion programs in high school education settings [e.g., reciprocal teaching style (Johnson and Ward, 2001; Mouratidou et al., 2007)]. However, these approaches can be limited by the use of hypothetical moral dilemmas and methodological constraints affecting engagement, motivation and involvement.

### Socio-Moral Reasoning Assessment and Intervention

There is a rich history of social cognition and competence assessment, however, recent recommendations aimed at enhancing the validity and ecology of such assessments highlight obstacles associated with traditional tasks such as the use of written, static and hypothetical scenarios (Beauchamp, 2017). Such approaches often introduce perceptual and cognitive confounds, do not adequately mimic the complexity and dynamism of real-life social scenarios, and limit user engagement, motivation, familiarity, presence, and immersion (Beauchamp, 2017; Morasse et al., 2021). A range of SMR assessment tools exist, including paper-and-pencil questionnaires, interviews and static cartoon presentation (Dooley et al., 2010). More recently, efforts have been deployed to increase the visual and dynamic nature of SMR assessment using pictures of real people in developmentally appropriate and realistic scenarios (Chiasson et al., 2017) and using virtual reality (Morasse et al., 2021).

### Serious Video Games

Serious video games, defined as “video games that use computer-based entertainment technology to teach, train, or change behavior” (Baranowski et al., 2008) are another potential medium for the assessment and remediation of socio-cognitive skills. They have already proven useful in the socio-cognitive domain, such as to improve emotion recognition (Silver and Oakes, 2001; Lacava et al., 2007) and social competence (Beaumont and Sofronoff, 2008) in youth with Autism Spectrum Disorders, or to improve executive functions in adolescents with Attention Deficit Hyperactivity Disorder (Dovis et al., 2015). The appeal of this method is a result of the popularity of recreational video games, their interactive nature, and their dynamic and adaptive qualities, which can promote youth engagement, stimulate affective reactions and boost motivation (Baranowski et al., 2008). This technology tends to elicit a sense of presence from players, defined as “the extent to which one feels present in the mediated environment, rather than in the immediate physical environment” (Steuer, 1992). It has been shown to correlate positively with empathy (Bachen et al., 2016; Morasse et al., 2021), and may contribute to participants feeling more immersed and therefore more engaged. Serious video games could therefore provide an innovative and engaging modality for assessing and optimizing SMR in adolescents.

### Objectives

The overarching objective of this study was to provide preliminary information on the development of a novel serious video game (MorALERT) for assessing and optimizing SMR in adolescence. Preliminary data are presented documenting SMR progression during an evaluative (EV) and an adaptive version (AV) of the game. The main difference between the versions is that the latter includes real-time assessment of SMR, automated scoring, and feedback and reinforcements that were directly tailored to responses provided. We hypothesized that players who completed the adaptive version, incorporating feedback and reinforcements in real-time, would improve their SMR throughout the game, but that players who completed the evaluative version, more comparable to a previously validated computerized task (SoMoral), would not. A secondary aim was to document the association between SMR, empathy, and sense of presence in adolescents who completed the game. It was expected that participants with higher empathic tendencies would have greater socio-moral maturity (i.e., higher SMR scores), and that those who felt most immersed in the game would show greater SMR and empathy.

## Methods

### Participants

Fifty-seven participants (27 females) between the ages of 12 to 17 years (*M* = 14.4, *SD* = 1.4 years) were recruited *via* community web sites and youth organizations (e.g., sports groups, clubs). For inclusion, participants had to be fluent in French and be enrolled in a regular school curriculum without having repeated a grade. Participants were excluded if they had a diagnosis of any neurodevelopmental, genetic, psychiatric or metabolic disorder or history of acquired brain injury.

### Procedure

Written consent was obtained from participants or their legal guardian. Participants completed either the EV (*n* = 24) or AV (*n* = 33) SMR serious video game MorALERT. Recruitment was conducted in two phases in parallel with developments in the game design itself. The first iteration of the game that was developed was the EV. A second development phase was subsequently initiated to develop the AV, thus recruitment occurred in sequence. For both groups, the assessment session included first a pre-test evaluation of SMR using three socio-moral dilemmas from a validated task (SoMoral, described below) to document participants’ initial SMR level. They then played the video game (MorALERT) and finally performed a post-test SMR assessment again using three dilemmas from the SoMoral. To complete the SoMoral and MorALERT, participants were seated at a desk in front of a desktop or laptop computer and were provided headphones to hear the audio stimuli. Standard instructions for both tests were provided and participants completed them on their own with no further input from the examiner. Responses were thus documented using audio recordings. Questionnaires documenting socio-demographic characteristics, empathy, and presence, as well as a brief intellectual functioning assessment were performed after the SMR assessment and game.

### Measures

#### Demographic Questionnaire

An in-house questionnaire was used to document age, sex, ethnicity, academic level, and parental education.

#### Intellectual Functioning

The two-subtest version (Vocabulary and Matrix Reasoning) of the Wechsler Abbreviated Scale Intelligence (WASI; Wechsler, 1999) was used to estimate general intellectual functioning (IQ, *M* = 100, SD = 15) for descriptive purposes.

#### Socio-Moral Aptitude Level Task

Six dilemmas from this previously validated task were equally divided into a pre- and a post-test to assess SMR progression before and after playing the video game. Detailed information on the SoMoral task, cognitive and affective factors associated with performance on the task, and performance in typically developing and clinical samples are presented elsewhere (Dooley et al., 2010; Beauchamp et al., 2013; Vera-Estay et al., 2016; Chiasson et al., 2017). Briefly, the SoMoral is a computerized task composed of everyday, visual socio-moral dilemmas each depicted by three static pictures. An initial screen represents the name of the dilemma, the next three screens correspond to pictures of real actors playing out a social situation (i.e., a problem associated with justice, welfare, harm or human rights) in the first-person perspective. Participants are asked whether or not they would engage in the action portrayed (moral decision-making) and asked to provide a justification for their response. For example, one of the dilemmas presents a scene during which the participant is losing at a game and must reflect on whether or not they would cheat to get ahead in the game. After viewing the three pictures, the participant is shown a screen that asks them what they would do in this situation (decision-making) and why (justification). In this study, participants’ justifications to the second question were recorded using a microphone function on the computer. The answers were then transcribed verbatim and the justifications were coded to obtain a SMR score using a cognitive-developmental approach with a score from 0 (no justification provided) to 5 points (highest level of socio-moral maturity). Lower scores are qualified by responses defined by egocentrism and fear of authority (e.g., I would not steal in a store because I could go to jail) and higher scores embody fundamental societal values such as people’s rights to property and integration of diverse points of view (e.g., the shopkeeper depends on selling his things and if people take things from him, he won’t have any money). For further details on the different stages and scoring system, please see Table 1 in Chiasson et al. (2017).

**TABLE 1:** Participants’ sociodemographic characteristics.

Characteristics	Valid N	Mean or N	SD or%	Statistical comparison between groups
Age	57	14.4	1.4	*t*(56) = 0.34, *p* = 0.73
Sex	57			Fisher’s Exact Test odds ratio = 1.59, *p* = 0.43
Female		27	47.4	
Male		30	52.6	
Ethnic background	48			χ*^2^*(5) = 6.35, *p* = 0.27
North America		14	24.6	
Europe		5	8.8	
Asia		15	26.3	
North Africa and Middle East		3	5.3	
Latin America		6	10.5	
Subsaharan Africa		5	8.8	
Education level (father)	39			χ*^2^*(4) = 2.91, *p* = 0.57
Elementary		0	0.0	
High school		4	7.0	
Cegep (college)		9	15.8	
Bachelor’s		16	28.1	
Master’s		6	10.5	
Doctorate		4	7.0	
Education level (mother)	43			χ^2^(5) = 7.15, p = 0.21
Elementary		1	1.8	
High school		4	7.0	
Cegep (college)		8	14.0	
Bachelor’s		17	29.8	
Master’s		11	19.3	
Doctorate		2	3.5	

#### MorALERT Serious Video Game

Two versions of the MorALERT video game were developed with Unity software and programmed in C# and Python languages. The game is played on a standard desktop computer and is in the third person perspective: the player incarnates a character whose avatar is visible on the screen. The player can choose the gender of the character in order to facilitate self-identification. Navigation in the virtual environment is performed by using the arrows of the keypad and all of the other actions are performed with the left click on the computer mouse.

##### Evaluative Version

**FIGURE 1:**
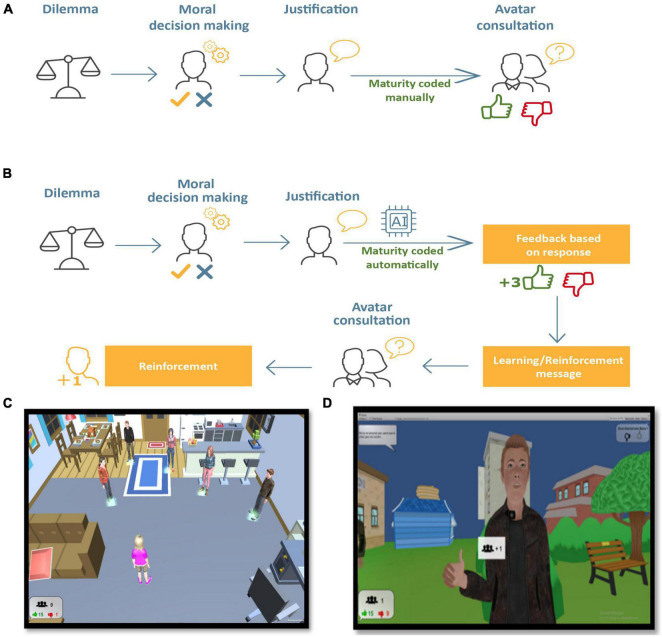
Schematic representation of the sequence for one dilemma in the MorALERT game. **(A)** Evaluative version: the structure and coded are comparable to the original SoMoral task. Coding of justifications is performed manually. Players can consult with avatar friends to hear how they reason and indicate whether they agree (thumbs up) or disagree (thumbs down) with their reasoning, however, no feedback or reinforcement is provided. **(B)** Adaptive version: The overall structure of the dilemmas is comparable to the original SoMoral task; however, justifications are coded automatically in order to provide tailored feedback in the form of “likes” and audio learning/reinforcement messages based on moral maturity stage. After consulting with avatar friends to hear their reasoning about the dilemma, players and indicate whether they agree (thumbs up) or disagree (thumbs down) with their reasoning and obtain “friends” when they agree with a moral reasoning stage that is comparable or higher than their own. Screen captures from the MorALERT game. **(C)** The player, seen in the third-person perspective, faces five avatar friends. In both the evaluative and AVs, using the keyboard arrows, the player can consult each of the friends who will provide their own reasoning to the dilemma in the form of audio sound files. **(D)** In the AV, when the player agrees with friend justifications that are equal or higher maturity than their own response, they gain a “friend.” The left bottom screen shows the number of friends and number of likes and dislikes accumulated in the game.

This version of the game is composed of nine everyday socio-moral dilemmas presented in a predetermined, continuous sequence (see Figure 1A). The scenes are dynamic as opposed to those in the SoMoral task which present static pictures and are presented in the third-person perspective. In each dilemma, players encounter the same five avatars (non-player characters, NPC) representing people they know (e.g., friends, classmates, and family) playing out realistic social situations with a moral component (see Figure 1C). The scene is also narrated using an audio track. For example, in one scene, the player avatar walks down the street behind someone who drops their wallet. After the wallet has fallen, a voice over integrated in the game asks the player to choose what they would do in this situation (decision-making) by clicking on one of two options (e.g., whether to keep the wallet or not). They are then asked to verbally justify their decision and to record their justification using the microphone function. Then, the five NPC appear and the player can approach them and interact with them. As the player nears a character, they provide their perspective on the socio-moral dilemma presented. Each NPC perspective represents one of the five stages of socio-moral maturity from the SoMoral coding system. The player indicates whether they agree or not with the NPC’s point of view by choosing a thumbs up or thumbs down. After each dilemma, a random score consisting of “likes” (a thumbs up symbol similar to that used on social media) and a tally of the player’s number of friends is shown to the player in the bottom left of the screen to encourage them to continue the game; these are not, however, graded according to their individual responses. After playing the game, the player’s justifications are transcribed verbatim and coded using the So-Moral coding system to obtain a SMR score corresponding to their level of socio-moral maturity.

##### Adaptive Version

This version of the game is similar to the EV in that they both have the same visual presentation, nine socio-moral dilemmas, and interactions with NPC avatars. The main difference is that the AV relies on an automated coding algorithm based on natural language processing, deep learning and expert knowledge from which an immediate SMR maturity score is produced as players express their moral justifications *via* microphone to a given dilemma (see Figure 1B). The algorithm was developed based on manually coded justifications provided in previous work using the SoMoral task; technical information on the design and reliability of the algorithm are available elsewhere (Tato et al., 2017, 2019).

In the AV of the game, this real-time scoring is used to trigger feedback and reinforcements to players throughout the game that are adapted to their decisions and justifications. Feedback and reinforcements are provided in three ways:

(i) Social feedback: the player receives “likes” from the NPC corresponding to the level of SMR maturity provided in their justifications. These are attributed according to the principal of rewarding more mature reasoning, but not penalizing lower stage responses (stage 1 = 5 likes, stage 2 = 8 likes, stage 3 = 11 likes, stage 4 = 14 likes, and stage 5 = 17 likes).
(ii) Learning messages: When players reach a new stage of socio-moral maturity, a praise message is shown on the screen in a dialog window as well as a message reflecting the essential elements of the reasoning stage. For example, the first time they reach Stage 3, they receive this message: “*Well done! You thought to consider others in making your decision*!” A learning message specific to the dilemma is also shown at the beginning of each dilemma in order to provide a clue for reaching the next stage of moral maturity. For instance, players whose response scored at Stage 2 in a dilemma about keeping a lost wallet would receive this message: “*Did you consider that the owner would like to have her wallet back*?”
(iii) Social reinforcements: as in the evaluative version, the adaptive design includes interactions with five avatar friends (NPC) in which players agree or disagree with their justifications using a thumbs up/thumbs down button. This process is also coupled with reinforcement mechanisms. If the player agrees (thumbs up) with a NPC’s justification that is equal or superior to their own stage of SMR maturity, they receive a friend icon. If participants disagree (thumbs down) with a superior or equal stage of sociomoral maturity, the NPC makes a thumbs down as a form of deterrent and the player does not receive a friend icon. These reinforcements accumulate in a box in the bottom, left corner at the bottom of the computer screen (see Figure 1D). Similarly, if the player reacts positively toward an inferior sociomoral maturity stage, they do not receive a friend icon and there is no reaction from the NPC.

#### Interpersonal Reactivity Index

The French adaptation (Gilet et al., 2013) of the IRI (Davis, 1980) was used to document empathy using 28 items (e.g., I am moved by the events that I witness) for which participants rate their empathy on a 5-point scale from 1 (Does not describe me well) to 5 (Describes me very well). The scale contains four 7-item subscales: (1) perspective taking scale, the tendency to adopt the point of view of other people (2) fantasy scale, the tendency of the respondent to feel the actions and feelings of fictive characters in books, movies and plays (3) empathic concern scale, measuring the feelings and worries in reaction to the other’s misfortune (4) personal distress scale, assessing personal feelings of anxiety and discomfort in interpersonal contexts. These items can be combined to obtain a global score (global IRI), an affective empathy subscore (affective IRI) and a cognitive empathy subcscore (cognitive IRI). The instrument’s reliability (Cronbach’s alpha between 0.67 and 0.87) and construct validity are adequate (Hawk et al., 2013).

#### Presence

The French version of the ITC-Sense of Presence Inventory (ITC-SOPI; Lessiter et al., 2001) was used to measure the individual experience of the participants while playing the game. The ITC-SOPI contains 44 items composed of a five-point Likert scale ranging from ″strongly disagree″ to ″strongly agree″. The scoring of the ITC-SOPI results in a total score encompassing four different factors of presence: Spatial presence, Engagement, Ecological Validity/Naturalness and Negative Effects.

### Statistical Analyses

Statistical analyses were performed using the RStudio software (version 1.4.1103). Despite the sequential design, the participant groups (EV and AV) were nonetheless compared on socio-demographic characteristic to ensure general comparability of the samples for descriptive purposes. *T*-tests were used to compare age and IQ, Fisher’s Exact Test was used to compare sex and Chi-squared tests were used to compare ethnic background and parental education. Given the non-randomized group attribution and preliminary nature of the study design, direct group comparisons on the main outcome (SMR) were not performed using mixed ANOVA. Instead, to document changes in socio-moral maturity, repeated measures ANOVAs were performed between the pre, in-game and post SMR maturity ratings for the EV and AV groups separately. To prevent sphericity issues, a Huynh-Feldt correction was applied to the obtained *p*-value (p_HF_). Bonferroni *post hoc* tests were used to determine where differences occurred between the pre-, in game and post-test SMR. Finally, correlations were performed to test the associations between in-game SMR, empathy (global IRI, affective IRI, and cognitive IRI) and sense of presence (total ITC). For these comparisons, the results of participants from both game versions were combined given the absence of *a priori* hypothesis pertaining to differences in these associations between game versions. Of note, nine participants (all in the Evaluative group) had missing data for the IQ or IRI measure due to the later inclusion of these measures in the study design.

## Results

### Sociodemographic Characteristics

Participants’ sociodemographic characteristics are presented in Table 1. Their average age was 14.40 years and they were from diverse backgrounds (26% Asian, 25% North American, 11% Latin American). The two groups (EV and AV) did not differ significantly in terms of sex (Fisher’s Exact Test odds ratio = 1.59, *p* = 0.43), age (*t*(56) = 0.34, *p* = 0.73), IQ (*t*(42) = −1.51, *p* = 0.14), ethnic background (χ*^2^*(5) = 6.35, *p* = 0.27), paternal (χ*^2^*(4) = 2.91, *p* = 0.57), and maternal (χ*^2^*(5) = 7.15, *p* = 0.21) education level.

### Socio-Moral Reasoning Progression Between Pre-test, In-Game, and Post-test

**FIGURE 2:**
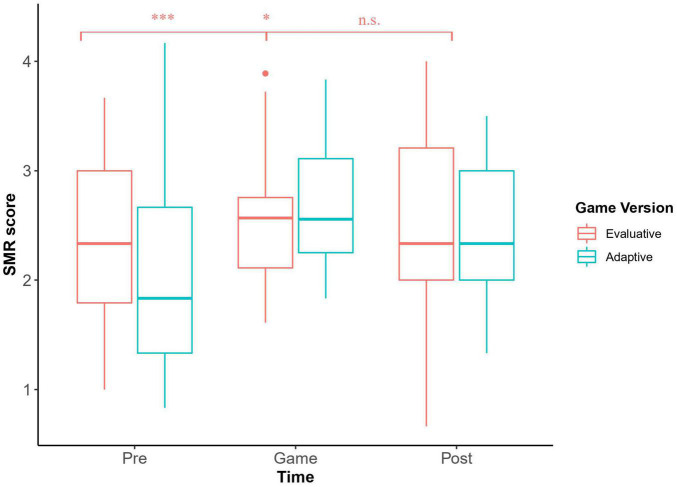
SMR score averages before, during and after the gameplay. As shown above, the average score of the adaptive group significantly increased from pre to in-game and that improvement was maintained to post, whilst the evaluative group’s average score stayed relatively stable throughout the experiment.

Socio-moral reasoning scores for the pre, in-game and post-test are presented in Table 2 along with the results of the secondary outcome measures (IRI, ITC). Repeated measures ANOVAs indicate that those who played the EV version did not improve their SMR (*F*(2,46) = 0.88, *p* = 0.42), while those who played the AV showed a significant improvement in SMR scores, with a strong effect size (*F*(1.97,63.00) = 9.81, *p_HF_* < 0.001, ϵ^2^ = 0.21) (Cohen, 1988). A *post hoc* Bonferonni test showed that in the AV version, significant differences were found between the pre and in-game SMR (*t*(64) = −4.39, *p* = 0.0001) as well as between the pre and post SMR scores, (*t*(64) = −2.68, *p* = 0.028). No significant difference was found between the in-game and post-test scores, (*t*(64) = 1.71, *p* = 0.21). The visual representation of those results can be see in Figure 2.

**TABLE 2:** Age and results on measures of interest according to group (evaluative and adaptive versions of MorALERT).

	Evaluative version				Adaptive version			
Scores	M	SD	Range	n	M	SD	Range	n
Age	14.46	1.35	12.00—17.00	24	14.33	1.36	12.00—17.00	33
IQ	104.67	10.07	89.00—128.00	15	110.0	10.39	89.00—123.00	28
ITC-SOPI	3.91	0.79	2.25—5.50	24	3.93	0.67	2.38—5.00	31
Pre-test SMR (So-Moral)	2.36	0.77	1.00—3.67	24	2.04	0.90	0.83—4.17	33
In-Game SMR (MorALERT)	2.57	0.58	1.61—3.89	24	2.67	0.56	1.83—3.83	33
Post-test SMR (So-Moral)	2.52	0.87	0.67—4.00	24	2.42	0.65	1.33—3.50	33
IRI-Global	3.42	0.39	2.68—3.82	15	3.33	0.49	2.00—4.07	32
IRI-Affective	3.26	0.31	2.71—3.71	15	3.10	0.47	1.79—4.00	32
IRI-Cognitive	3.57	0.60	2.43—4.36	15	3.57	0.63	2.00—4.71	32

*IRI, Interpersonal Reactivity Index; ITC-SOPI, International Test Commission-Sense of Presence Inventory; SMR, Socio-moral reasoning.*

### Correlations Between Socio-Moral Reasoning, Empathy and Presence

Associations between the in-game SMR scores and empathy neared significance for the global IRI empathy score (*r* = 0.26, *p* = 0.07), and the cognitive IRI empathy subscore (*r* = 0.28, *p* = 0.06) with a medium effect size (Cohen, 1988). No significant correlations were found between SMR and sense of presence (ITC; *r* = 0.17, *p* = 0.21), between global empathy and sense of presence (*r* = 0.16, *p* = 0.31), between cognitive empathy and sense of presence (*r* = 0.06, *p* = 0.69) and between affective empathy and sense of presence (*r* = 0.24, *p* = 0.11).

## Discussion

The main objective of the study was to provide preliminary information on the development of a novel serious video game for assessing and optimizing SMR in adolescence and on the effect of evaluative and adaptive versions of the game. The results support the main study hypothesis in that adolescents who played an AV of the game, which incorporated feedback and reinforcement message in the form of learning and motivation cues, significantly improved their SMR by playing the game, while those who played an evaluative version (no feedback or other reinforcements) did not. However, the sequential and non-randomized study design precluded direct comparisons between the two versions of the game and a more controlled experimental design is necessary to draw clear conclusions as to the relative value of the two versions. Nonetheless, the study results provide initial information on this novel approach to social cognition assessment and optimization. Contrary to expectations, no significant relations were found between SMR, empathy and sense of presence.

### Efficacy of MorALERT

The main study findings provide preliminary support for the potential of a serious video game to assess and optimize SMR in adolescents. To our knowledge, this is the first video game that directly focusses on SMR skills. As such, there are no published studies to compare the findings with directly, however, game-like media have previously been used to target other sociocognitive skills. Our results align with studies reporting improvements in affect recognition (emotional regulation, recognition and expression) in children with ADHD using the *EmoGalaxy* serious video game (Hakimirad et al., 2019) and in neurotypical children with the *Socialdrome* serious video game (Tan et al., 2016).

On the surface, the two versions are similar since the same dilemmas are presented; however, the presence of real-time feedback and reinforcements that are tailored to the player’s responses distinguishes the learning process experienced by participants who played the AV. Two well-established psychological principles could explain the SMR improvement observed in the AV: operant conditioning (Skinner, 1938) and the effect of personal relevance (Sorrentino et al., 1988). Operant conditioning effects are likely to be present in association with the reward “likes” offered to players when they provide answers of progressively higher moral maturity stages. The rewarding or distressing effect of social media-type likes and friend counts have been extensively reported in other contexts (Sherman et al., 2016, 2018; Lee et al., 2020) and, in the game, serve a similar reinforcement purpose. Notably, the game included only positive reinforcements. These were given when the target behavior was elicited, that is, providing reasoning at a higher moral stage than in the previous dilemma. No punishments or negative reinforcements were included.

In terms of personal relevance, a significant procedural and design difference exists between the EV and AV of the game. The latter integrated an automated coding algorithm that allowed us to provide personally tailored advice to players throughout the game, and this may have made them feel more involved and heightened the relevance and salience of the dilemmas. Empirical work indicates that the more people feel personally involved in situations presented to them, the more they exhibit strong emotional reactions rather than remaining in a theoretical mindset (Darley and Lim, 1992). Inclusion of effective real-world social reinforcements throughout the game to elicit authentic emotional reactions, as well as the presentation of social scenarios typically experienced or encountered by youth bodes well for establishing both internal and ecological validity in future work. The findings on the AV also offer some support and promise in terms of the feasibility of using artificial intelligence within a social cognition tool, as a way to rapidly and accurately code SMR maturity online, in real-time, and bodes well for further applications in future versions of the game or other interactive or digital technologies.

### Associations Between Socio-Moral Reasoning, Empathy and Presence

Associations between SMR, empathy and presence were not supported, though the correlations between SMR and empathy showed a trend toward a positive relation and may have been limited by the modest sample size. Some studies have found significant links between these two constructs (Hoffman, 2001; Nicovich et al., 2005; Barriga et al., 2009; Dooley et al., 2010; Bachen et al., 2016; Morasse et al., 2021). With regard specifically to the SoMoral, previous work by our group has reported equivocal findings in this regard. As in the current study, Vera-Estay et al. (2016) found only a near significant relation between SMR and cognitive empathy; however, Morasse et al. (2021) did find a significant relation when a virtual reality version of the SoMoral was tested and interpreted as being due to the more immersive nature of the task. Notably, MorALERT, is interactive, but not immersive. However, it is possible that players completing the AV may feel a greater sense of presence because of the heightened interactive component (feedback) compared to the EV. In this study, effects of presence and personal relevance may overlap or be confounded. Presence is defined as “the extent to which one feels present in the mediated environment, rather than in the immediate physical environment” (Steuer, 1992), while personal relevance is related to how much someone recognizes themselves in a situation and how relevant the situation is to their goals and values (Celsi and Olson, 1988). However, it is possible that those for whom the situation is most relevant also feel more present. Future work using a larger sample and simultaneous randomized to the two versions would allow for direct comparisons between the versions on presence, personal relevance, and other variables of interest.

Another reason for the lack of SMR-empathy link in the current study could be methodological differences in the presentation of the social scenarios. The original SoMoral consists of first-person perspective pictures, whereas the serious video game is played from a third-person perspective, which could make it more difficult for some participants to feel present and engaged when playing the game. Perspective-taking manipulation studies show that the affective processes underlying empathy are more exploited from a first-person perspective than from a third-person perspective (Jackson et al., 2006; Lamm et al., 2007). This increased distance between the player and the characters could dampen their ability to empathize with the avatars in the game.

### Strengths, Limitations, and Future Directions

To our knowledge, this is the first serious video game designed to assess and optimize SMR in adolescents and is grounded in previous empirical work and validation studies in both neurotypical children and adolescents and those with acquired or neurodevelopmental disorders. The results, however, need to be considered in light of a number of limitations. First, this is an initial step in the development and study of a serious video game and the sample size is modest and may have limited the detection of some associations between variables. Second, the study was not designed as a randomized control intervention trial and though the samples were comparable in terms of age and IQ, they were recruited sequentially limiting the possibility of direct comparisons between the two. Third, empathy data was only available for a subset of the sample and most of the participants who completed the empathy measure were from the adaptive group. Fourth, we did not collect information on socio-moral decision-making although this variable does exist in the original SoMoral task. However, in previous studies in typically developing children and adolescents, very few individuals made maladaptive decisions and it is likely that this score is subject to social desirability, thus this variable was not useful in characterizing performance. Significant changes in moral decision-making are, however, observable in some clinical populations, such as for example youth with Traumatic Brain Injury (Beauchamp et al., 2019). Thus, it is not clear in the current study how the participants’ reasoning relates to their social decisions and behavior.

Future avenues of research should establish the psychometric properties of MorALERT and test its effects in a large sample, as well as verify which affective, cognitive, social, individual (e.g., learning styles, temperament, traits) and behavioral factors contribute to success in the game, or conversely, impede performance. It is possible that other methods of social learning may be comparable in effect to this video game. Using a longitudinal intervention design with children randomized to each of the video game conditions in addition to a more traditional, low-tech learning control condition could inform on the potential added value of the gamified approach and would allow for direct comparisons between experimental and control conditions, as well as verification of knowledge retention effects in the longer term. Finally, further methodological developments could explore differences in user perspective and test more immersive formats such as augmented or virtual reality for heightening engagement.

## Conclusion

Serious video games offer an interesting avenue for quantifying and remediating social competence in typically developing youth at-risk. Gamifying knowledge acquisition can heighten learning (Vogel et al., 2006; Tüzün et al., 2009; Miller and Robertson, 2010) and constitutes a motivating medium for youth. The findings of this study using a serious SMR video game show promise in terms of its potential for assessing and possibly improving moral maturity, though further work both in terms of game design and empirical validation are necessary.

## Data Availability Statement

The datasets presented in this article are not readily available because Consent was not obtained from the parents to share the data. Requests to access the datasets should be directed to MB, miriam.beauchamp@umontreal.ca.

## Ethics Statement

The studies involving human participants were reviewed and approved by University of Montreal Research Ethics Commitee. Written informed consent to participate in this study was provided by the participants’ legal guardian/next of kin.

## Author Contributions

JL-M, AT, EV-E, SC, AD, RN, and MB contributed to the study conception and design. MB, AD, and RN contributed to the development of the study hypotheses. JL-M, AT, AL-B, and AB performed the testing and data collection. HZ performed the data analysis and interpretation under the supervision of MB. HZ drafted the manuscript with input from MB. All authors read, revised and approved the final version of the manuscript for submission.

## Conflict of Interest

The authors declare that the research was conducted in the absence of any commercial or financial relationships that could be construed as a potential conflict of interest.

## Publisher’s Note

All claims expressed in this article are solely those of the authors and do not necessarily represent those of their affiliated organizations, or those of the publisher, the editors and the reviewers. Any product that may be evaluated in this article, or claim that may be made by its manufacturer, is not guaranteed or endorsed by the publisher.
